# Antigen-Independent IFN-γ Production by Human Naïve CD4^+^ T Cells Activated by IL-12 Plus IL-18

**DOI:** 10.1371/journal.pone.0018553

**Published:** 2011-05-10

**Authors:** Rachel B. Munk, Katsuki Sugiyama, Paritosh Ghosh, Carl Y. Sasaki, Louis Rezanka, Kasturi Banerjee, Hidenori Takahashi, Ranjan Sen, Dan L. Longo

**Affiliations:** 1 Lymphocyte Cell Biology Unit, Laboratory of Molecular Biology and Immunology, National Institute on Aging, National Institutes of Health, Baltimore, Maryland, United States of America; 2 Gene Regulation Section, Laboratory of Molecular Biology and Immunology, National Institute on Aging, National Institutes of Health, Baltimore, Maryland, United States of America; National Institutes of Health - National Cancer Institute, United States of America

## Abstract

The role of T cells in innate immunity is not well defined. In this report, we show that a subset of human peripheral blood CD4^+^ T cells responds to IL-12 plus IL-18, but not to IL-12 or IL-18 alone, by producing IFN-γ in the absence of any antigenic stimulation or cell proliferation. Intracellular staining reveals a small percentage of resting CD4^+^ T cells (0.5 to 1.5%) capable of producing IFN-γ in response to IL-12 plus IL-18. Interestingly, both naïve (CD45RA^+^) and memory (CD45RO^+^) CD4^+^ populations were responsive to IL-12 plus IL-18 stimulation in producing IFN-γ. The expression of IFN-γinduced by IL-12 and IL-18 is sensitive to rapamycin and SB203580, indicating the possible involvement of mTOR and p38 MAP kinase, respectively, in this synergistic pathway. While p38MAP kinase is involved in transcription, mTOR is involved in message stabilization. We have also shown that NFκB family member, cRel, but not GADD45β and GADD45γ, plays an important role in IL-12 plus IL-18-induced IFN-γ transcription. Thus, the present study suggests that naïve CD4^+^ T cells may participate in innate immunity or amplify adaptive immune responses through cytokine-induced antigen-independent cytokine production.

## Introduction

Interferon γ (IFN-γ) plays an important role in host defense. The major sources of this cytokine are T cells and natural killer (NK) cells [1]. In T helper type 1 (Th1) cells, at least two distinct receptor-mediated pathways can induce IFN-γ production [2], [3]. Differentiated Th1 cells can produce IFN-γ upon T cell receptor (TCR) engagement, which is enhanced by co-stimulation through anti-CD28 and is sensitive to cyclosporin A [2], [3]. This TCR-induced IFN-γ production is antigen-specific, associated with T cell proliferation, and a component of the adaptive immune response. Also, differentiated murine Th1 cells that have acquired expression of both interleukin 12 (IL-12) and IL-18 receptors can directly produce IFN-γ in response to IL-12 and IL-18 in the absence of TCR engagement; both act synergistically to enhance IFN-γ production through pathways that are insensitive to cyclosporin A [2], [3]. This TCR-independent IFN-γ production is antigen-nonspecific and a component of the innate immune response.

The synergistic effect of IL-12 and IL-18 on IFN-γ production is not fully understood. One of the mechanisms that could partially explain the synergism of IL-12 and IL-18 for IFN-γ production is the reciprocal up-regulation of their receptors in responding cells [4], [5], [6]. Another mechanism is simultaneous activation and cooperation of STAT4, GADD45β, NF-κB, and/or AP-1 that induce IFN-γ transcription [3], [7], [8], [9], [10], [11]. In this report, we demonstrate that human peripheral blood CD4^+^ T cells produce IFN-γ after treatment with IL-12 and IL-18, but not with IL-12 or IL-18 treatment alone, and the IFN-γ production is sensitive to rapamycin and SB203580, indicating the possible involvement of mTOR and p38 MAP kinase. Interestingly, naïve and memory CD4^+^ T cells are responsible for IFN-γ production. Our results suggest that T cells may play a critical role in innate immunity through cytokine-induced antigen-independent cytokine production.

## Materials and Methods

### Cells and tissue cultures

Peripheral blood mononuclear cells (PBMC) collected from healthy donors were isolated by Ficoll-Paque density gradient centrifugation. Collection of blood from donors (who provided informed written consent) were done according to the protocol (#2003-054) approved by the NIA review board. CD4^+^ T cells were purified from PBMC using MACS CD4^+^ T cells isolation kit II (Miltenyi Biotec, Auburn, CA). The isolation kit contains a CD56 antibody, which in addition to NK cells, also depletes CD56^+^ T cells. Staining with surface markers routinely revealed greater than 98% CD4^+^ T cells. Naïve (CD45RA^+^) and memory (CD45RO^+^) T cells were negatively selected from CD4^+^ T cells either using CD45RO microbeads or CD45RA microbeads, respectively (Miltenyi Biotec). Purity of each cell subset was >98%. IL-18Rα^+^ cells were isolated from either CD45RA^+^ or CD45RO^+^ T cells by positive selection using anti-IL-18Rα-phycoerythrin monoclonal antibody (FAB840P; R&D Systems, Minneapolis, MN) followed by anti-PE Microbeads (Miltenyi Biotec). All the cells, except IL-18Rα^+^ cells were cultured in RPMI 1640 with 10% fetal bovine serum (FBS), 100 U/ml penicillin, 100 µg/ml streptomycin, and 2 mM glutamine. Autologous whole blood serum (10%) instead of FBS and IL-2 (10 units/ml) were used in culturing IL-18Rα^+^ cells. Resting cells were stimulated with 1 ng/ml of recombinant human IL-12 and/or 40 ng/ml of recombinant human IL-18 for various periods of time. Pretreatment with rapamycin and SB203580 was done 60 minutes before IL-12/IL-18 stimulation. Splenic CD4^+^ T cells were isolated from 8–12 week old mice using CD4^+^ isolation kit (Miltenyi Biotec). Murine cells were cultured in RPMI 1640 with 10% fetal bovine serum (FBS), 100 U/ml penicillin, 100 µg/ml streptomycin, 2 mM glutamine, 10 mM HEPES and 55 µM β-mercaptoethanol. Cells were stimulated with 1 ng/ml of recombinant murine IL-12 and/or 40 ng/ml of recombinant murine IL-18.

### Mice

C57/BL6 male mice of 8–12 weeks old were used for the experiment, and these mice were bred in the NIA animal facility according to the protocol (423-CMS-2013) approved by the Animal Care and Use Committee in the National Institute on Aging at the National Institutes of Health.

### Reagents

Recombinant human and murine IL-12 and murine IL-18 were purchased from R&D Systems (Minneapolis, MN). Recombinant human IL-18 was purchased from Medical & Biological Laboratories Co., LTD. (Woburn, MA). Actinomycin D and cycloheximide (CHX) were purchased from Sigma (St. Louis, MO). PE anti-human IFN-γ, FITC anti-human CD4, BD Cytofix/Cytoperm, BD Perm/Wash, and anti-phospho Stat4 (Y693) antibody were purchased from BD Biosciences (San Jose, CA). Anti-Stat4 antibody was purchased from Invitrogen (Carlsbad, CA). Monensin solution was purchased from BioLegend (San Diego, CA). Rapamycin, SB203580, cyclosporin A, IKK-2 inhibitor, SC-514, and ionomycin were purchased from Calbiochem (San Diego, CA).

### Measurement of IFN-γ production

CD4^+^ T cells were stimulated for 24, 48, and 72 hours and the supernatants were analyzed by Cytometric Beads Array (CBA) Kit (BD Bioscience, San Diego, CA) according to the manufacturer's instructions.

### Intracellular staining for IFN-γ

CD4^+^ T cells were stimulated for 48 hours with IL-12 plus IL-18 in the presence or absence of SB203580. Approximately 18 hours before cells were harvested, monensin solution (1×) was added to stop the secretion of cytokine. Cells were collected and washed with cold PBS. Cells were resuspended in 1 ml 10% human serum in PBS, and were incubated for 20 min at 4°C. Cells were collected and resuspended in 250 µl BD Cytofix/Cytoperm solution and incubated for 20 min at 4°C. Cells were washed two times with 1 ml BD perm/wash solution (1×). Solution was poured off, and cells were resuspended in the leftover solution. Cells were incubated with PE anti-human IFN-γ and FITC anti-human CD4 for 30 min at 4°C in the dark. Cells were washed twice with 1 ml BD perm/wash solution (1×), and resuspended in fixing solution before analyzing by Becton Dickinson FACSan.

### Statistical analysis

For statistical analysis, p-value was calculated by Student's t-test using one-tail paired analysis.

### Quantitative Real-time PCR

Total RNA was isolated by RNeasy Mini Kit (Qiagen, Valencia, CA). Complementary DNA (cDNA) was synthesized from 1 µg of total RNA using SuperScript III reverse transcriptase kit and random hexamers (Invitrogen, Carlsbad, CA) in a total volume of 20 µl. One-hundredth of the sample was used in a real-time PCR reaction containing a 0.2 µM concentration of both forward and reverse primers and iTaq SYBR Green Supermix with ROX (Bio Rad, Hercules, CA). Quantitation of fold induction was analyzed by the 2^−ΔΔCT^ method [12]. The quantity of each cDNA was normalized by GAPDH. The following PCR primers were used for amplification: IFN-γ, 5′- TGACCAGAGCATCCAAAAGA -3′ and 5′-CTCTTCGACCTCGAAACAGC -3′; GADD45β, 5′-GTGTACGAGTCGGCCAAGTT-3′ and 5′-TTGATGTCGT TGTCACAGCA-3′; GADD45γ, 5′-GGGTCCACGTTCAAGACTTT-3′ and 5′-GACACAGTTCCGGAAAGCAC-3′; glyceraldehyde-3-phosphate dehydrogenase (GAPDH), 5′- CGACCACTTTGTCAAGCTCA -3′ and 5′-AGGGGAGATTCAGTGTGGTG -3′; murine IFN-γ, 5′- TGAGCTCATTGAATGCTTGG-3′ and 5′-ACAGCAAGGCGAA AAAGGAT-3′; and murine β-actin-5′- GGGGTGTTGAAGGTCTCAAA-3′ and 5′- TGTTACCAACTGGGACGACA.

## Results

### Human peripheral blood CD4^+^ T cells are responsive to IL-12 plus IL-18 in producing IFN-γ in an antigen independent manner

**Figure 1:**
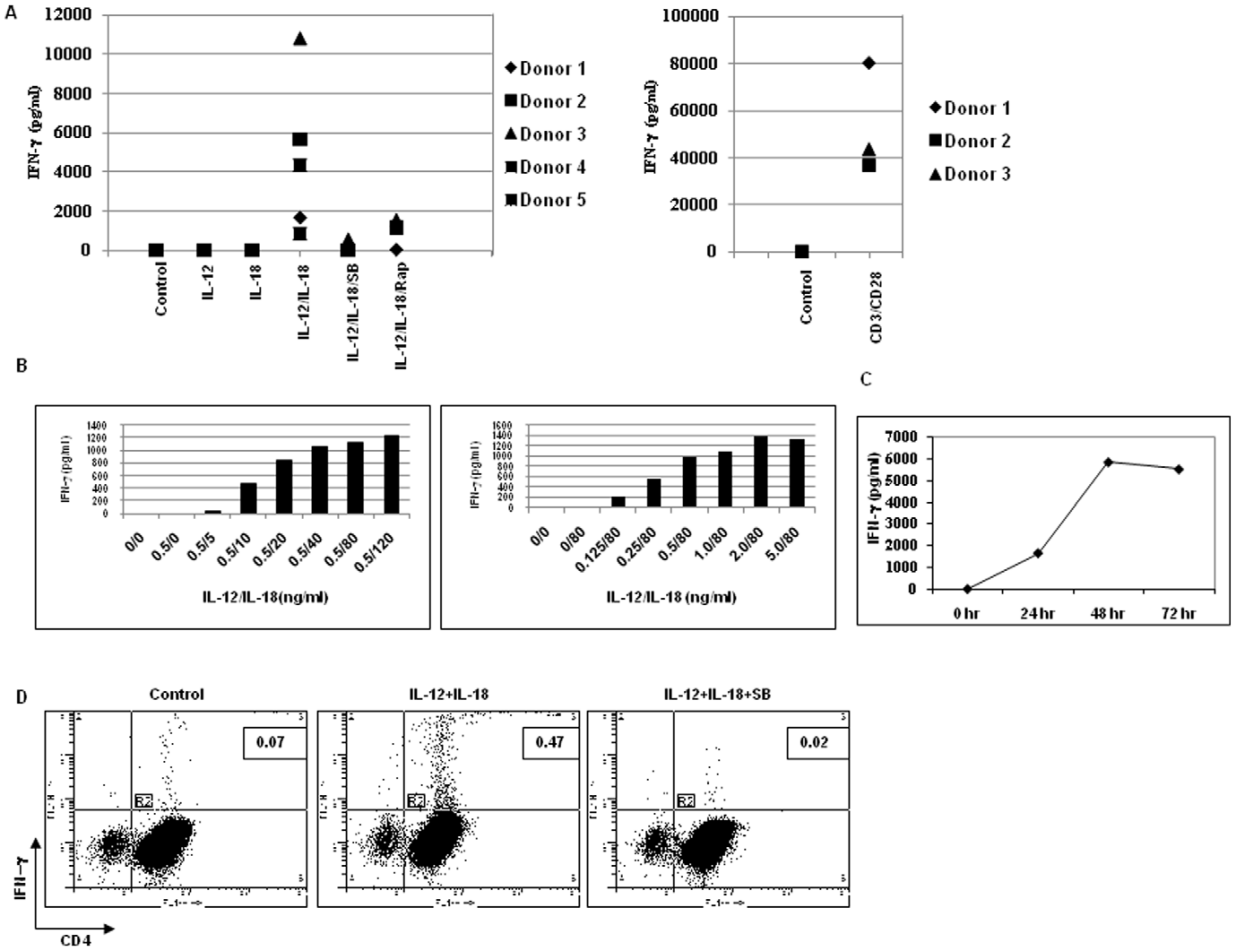
Production of IFN-γ by human peripheral blood CD4^+^ T cells after activation with IL-12 plus IL-18, but not with IL-12 or IL-18 alone. (A) Human peripheral blood CD4^+^ T cells were stimulated with IL-12 (1 ng/ml) and/or IL-18 (40 ng/ml) for 24 hours in the presence or absence of SB203580 (10 µM) and rapamycin (20 ng/ml). CD4^+^ T cells were also activated with cross-linked anti-CD3 (200 ng/ml) plus anti-CD28 (0.5 µg/ml) for 24 hours. Supernatants were analyzed for IFN-γ production by Cytometric Beads Array. (B) Peripheral blood CD4^+^ T cells were stimulated with the indicated concentrations of IL-12 and/or IL-18 for 24 hours and the supernatants were analyzed for IFN-γ production. Results from one representative donor out of two independent donors are shown here, and data from other donor are shown in Figure S2. (C) Peripheral blood CD4^+^ T cells were stimulated with IL-12 (1 ng/ml)/IL-18 (40 ng/ml) for various periods of times to measure IFN-γ production. Results from one representative donor out of three independent donors are shown here, and the results from other donors are shown in Figure S3. (D) Peripheral blood CD4^+^ T cells were activated with IL-12 plus IL-18 in the presence or absence of SB203580 for 48 hours, and the cells were then used for intracellular staining for IFN-γ. One representative donor among three is shown here, and the results from rest of the donors are shown in Table 1.

IL-12 and/or IL-18 can induce production of IFN-γ by differentiated Th1 cells, which have acquired expression of both IL-12 and IL-18 receptors. Recently, it has been shown that some populations of resting human T cells express IL-12 receptor β1 and IL-18 receptor α [13], [14]. We first examined whether IL-12 and/or IL-18 can induce production of IFN-γ by resting human peripheral blood CD4^+^ T cells. Highly purified CD4^+^ T cells were stimulated with IL-12 and IL-18, either alone or in combination, and in the presence or absence of either rapamycin, an mTOR inhibitor, or SB203580, a p38 MAP kinase inhibitor, for 24 hours. As shown in Figure 1A, only IL-12 plus IL-18, but not IL-12 or IL-18 alone, induced IFN-γ production by resting human peripheral blood CD4^+^ T cells. The amount of IFN-γ produced by five different donors ranged from 863.8 to 10,829.7 pg/ml after 24 hour stimulation. The production of IFN-γ by IL-12 plus IL-18 was sensitive to rapamycin and SB203580, but not to cyclosporin A (Fig. 1A & Supplemental Fig. 1A). The amount of IFN-γ produced in response to IL-12 plus IL-18 was substantially lower (48 fold) in comparison to the total amount produced by anti-CD3 plus anti-CD28 stimulation, and this activation-induced IFN-γ production was sensitive to cyclosporin A (Fig. 1A & S1). The activation with IL-12 and IL-18 had a synergistic effect on resting human peripheral blood CD4^+^ T cells in producing IFN-γ (Fig. 1B, and S2), and the synergism was increased with time (Fig. 1C, and S3). Although, the cytokine stimulation of resting CD4^+^ T cells induced IFN-γ production, this stimulation did not induce any proliferation or cell death (Fig. S4).

In order to determine the percentage of resting CD4^+^ T cells that were capable of producing IFN-γ upon IL-12 plus IL-18 stimulation, purified CD4^+^ T cells were activated with IL-12 plus IL-18 in the presence or absence of SB203580 for 48 hours, and the percentage of IFN-γ T cells was determined by intracellular staining. As shown in Fig. 1D, 0.47% of total CD4^+^ T cells were IFN-γ positive upon activation, which was a 6.7-fold increase as compared to untreated cells. This percentage decreased to the basal level in the presence of SB203580. Among three independent donors, the highest percentage of IFN-γ positive CD4^+^ T cells observed was 1.5% (Table 1). These data indicate that a small percentage of resting CD4^+^ T cells is capable of responding to IL-12 plus IL-18 treatment in producing IFN-γ.

**Table 1:** Percentage of IFN-γ ^+^ CD4^+^ T cells as determined by intracellular staining.

	Control	IL-12+IL-18*	IL-12+IL-18+SB**
Donor 1	0.07	0.47	0.02
Donor 2	0.05	1.5	0.07
Donor 3	0.04	0.8	0.02

*p = 0.05;

**p = 0.045.

### Effect of IL-12 plus IL-18 treatment on IFN-γ mRNA level

**Figure 2:**
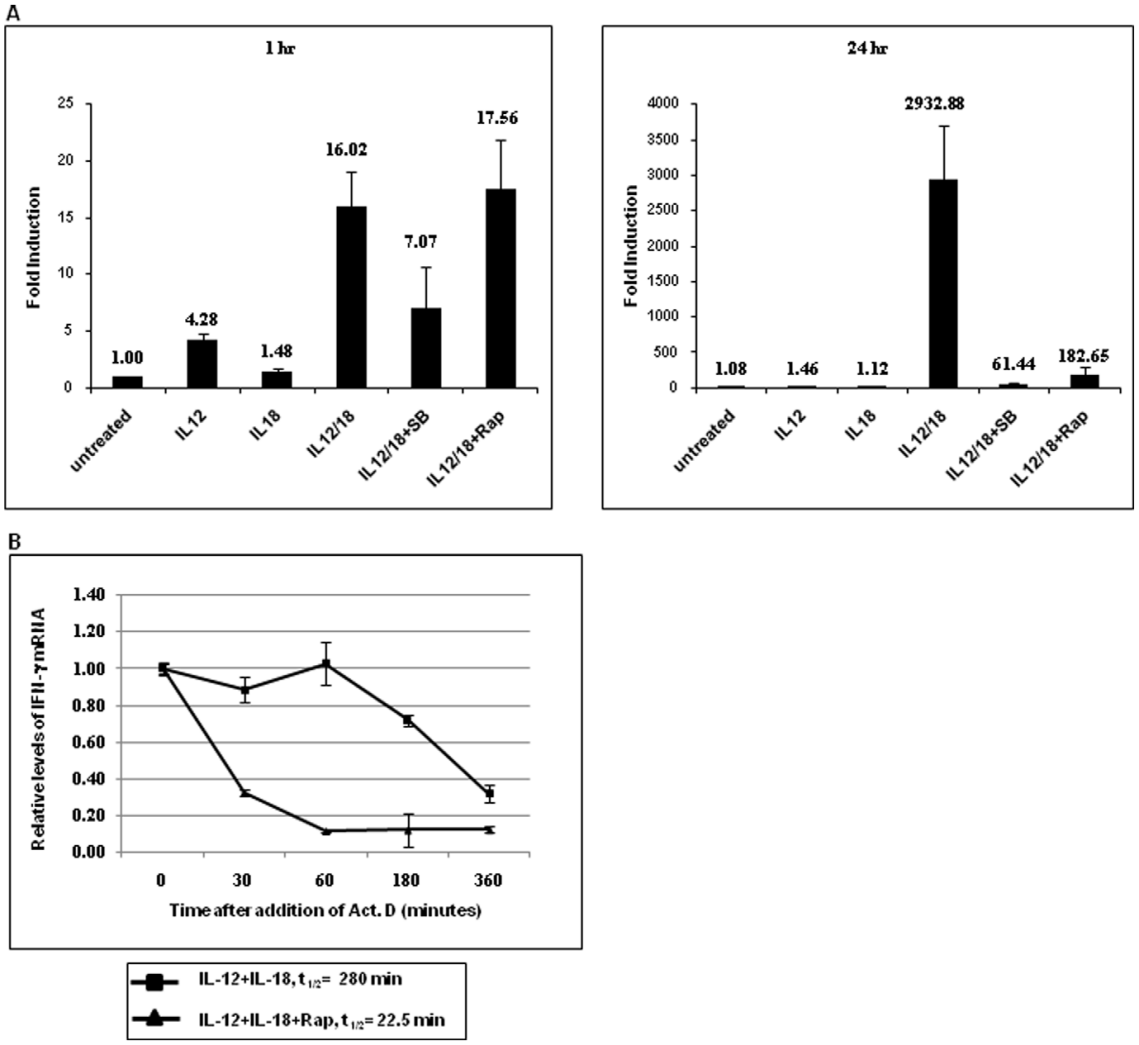
Effect of cytokine treatment on IFN-γ mRNA levels. (A) Human peripheral blood CD4^+^ T cells were stimulated with IL-12 and/or IL-18 for either 1 hour (left panel) or 24 hours (right panel) in the presence or absence of rapamycin or SB203580. Total RNAs were isolated and analyzed by real-time PCR. The quantity of each cDNA was normalized by GAPDH. Data represent average ± SD from triplicate. (B) Effect of rapamycin on IL-12 plus IL-18-induced IFN-γ mRNA stability. Peripheral blood CD4^+^ T cells were stimulated with IL-12 plus IL-18 for 18 hours in the presence or absence of rapamycin. At 18 hours, actinomycin D (1 µg/ml) was added to stop new RNA synthesis, and the cultures were incubated further for various periods of times. After each point, total RNAs were isolated and analyzed by real-time PCR. The quantity of each cDNA was normalized by β-actin. Data represent average ± SD from triplicate.

To determine the mechanism by which IL-12 plus IL-18 treatment increases IFN-γ production, human peripheral blood CD4^+^ T cells were treated with IL-12 and IL-18, either alone or in combination, and in the presence or absence of either SB203580 or rapamycin at two different time points (1 and 24 hours). Total RNAs were isolated and were analyzed for IFN-γ message by real-time PCR. As shown in Fig. 2A, IL-12 alone had some effect on the transcription of IFN-γ (4-fold induction) at the earlier time point, whereas the combination of IL-12 and IL-18 had a synergistic effect on the expression of IFN-γ mRNA (16-fold induction), which was partially blocked by SB203580. The synergistic effect was further increased with time (almost 3000 fold) after 24 hours of activation. When we tested the effect of rapamycin on the synergistic effect of IL-12 plus IL-18 treatment, no effect was observed at the earlier time point (Fig. 2A). At the later time point (24 hour treatment), rapamycin had a significant suppressive effect, indicating a possible role for mTOR in IFN-γ mRNA stability. To determine whether mTOR might be involved in regulating IFN-γ mRNA at the post transcriptional level, freshly isolated T cells were stimulated with IL-12 plus IL-18 for 18 hours in the presence or absence of rapamycin. At 18 hours, actinomycin D was added to stop new RNA synthesis, and the cultures were incubated further for various periods of times. After each point, total RNAs were isolated and analyzed by real-time PCR. As shown in Fig. 2B, the rate of decay for activation-induced IFN-γ mRNA levels post actinomycin D treatment was faster in rapamycin-pretreated samples as compared to IL-12 plus IL-18 treated samples (t_1/2_ = 22.5 vs 280 min., respectively). These results suggest that the synergistic effect of IL-12 plus IL-18 treatment on IFN-γ expression is a function of both transcriptional and post-transcriptional effects.

### Synergy between IL-12 and IL-18 signaling was observed in Stat4 phosphorylation

**Figure 3:**
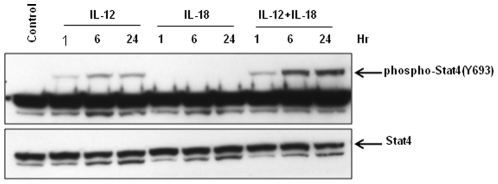
Synergistic effect of IL-12 plus IL-18 treatment on Stat4 phosphorylation. Human peripheral blood CD4^+^ T cells were treated with IL-12 and IL-18, either alone or in combination, for different periods of time. Equal amounts of whole cell lysates (35 µg) were analyzed by western blot analysis.

To investigate the signaling synergy between IL-12 and IL-18 stimulation, human peripheral blood CD4^+^ T cells were stimulated with IL-12 and IL-18, either alone or in combination, for different periods of time. Whole cell lysates were then analyzed for Stat4 phosphorylation by western blot analysis. As shown in Figure 3, the synergy between IL-12 and IL-18 signaling was observed as early as 1 hour, and the synergy was increased with time and persisted for at least 24 hours. These data suggest a possible mechanism underlying the requirement for both IL-12 and IL-18 stimulations in producing IFN-γ.

### IL-12 plus IL-18-induced IFN-γ mRNA level was independent of de novo protein synthesis, GADD45β and GADD45γ, but dependent on cRel

**Figure 4:**
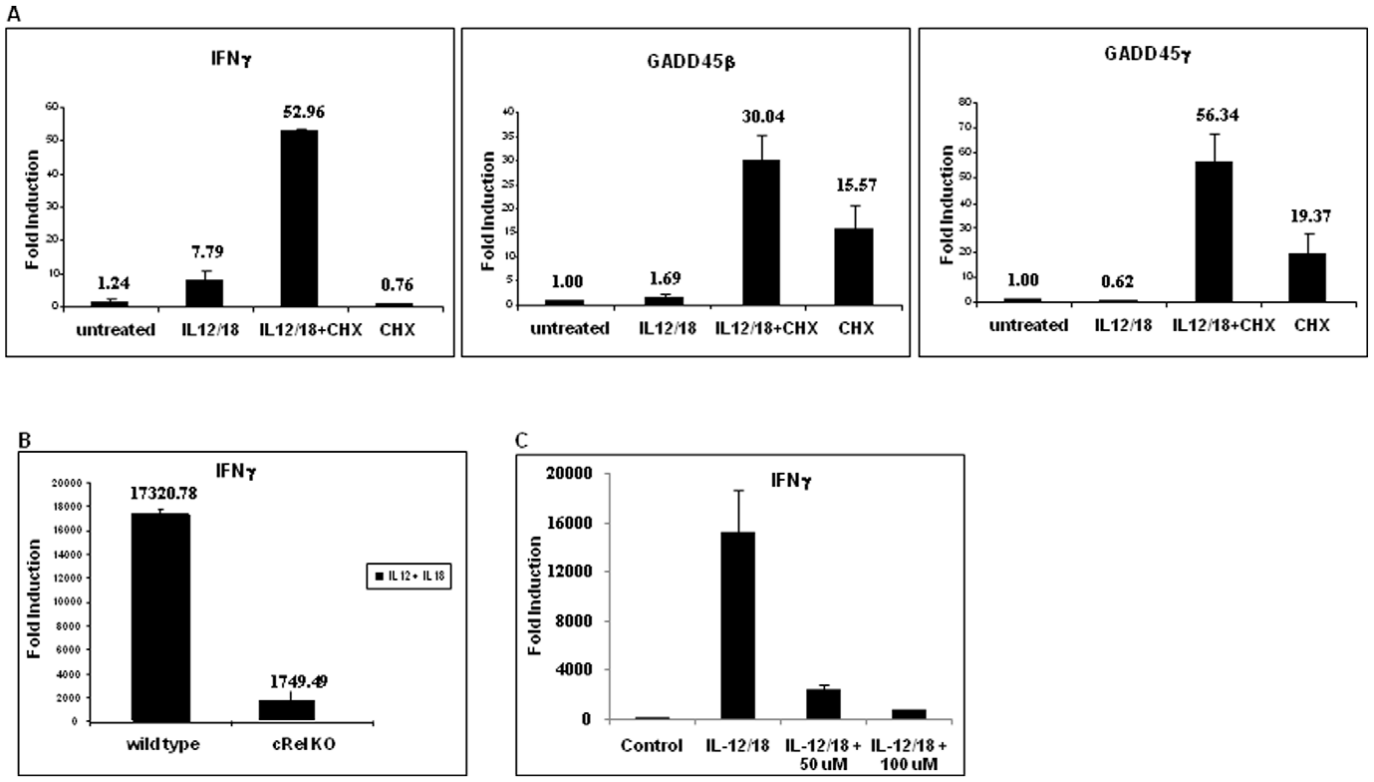
Effect of cytokine treatment on the mRNA levels of IFN-γ, GADD45β and GADD45-γ. (A) Human peripheral blood CD4^+^ T cells were pre-treated with cyclohexamide (10 µg/ml) for 30 minutes followed by stimulation with IL-12 and IL-18 for 4 hours. Total RNAs were isolated and analyzed by real-time PCR. The quantity of each cDNA was normalized by GAPDH. Data represent average ± SD from triplicate. (B) Role of cRel in cytokine signaling. Splenic CD4^+^ T cells from wild type and cRel knockout mice were activated with IL-12 plus IL-18 for 24 hours and the IFN-γ mRNA levels were determined by real-time PCR. The quantity of each cDNA was normalized by GAPDH. Data represent average ± SD from triplicate. (C) Role of NFκB/Rel family transcription factors in cytokine signaling in human CD4^+^ T cells. Human CD4^+^ T cells were pretreated with IKK-2 inhibitor (50 and 100 µM) for 1 hour before activating the cells with IL-12 plus IL-18 for 18 hours. IFN-γ mRNA levels were then determined by real-time PCR. The quantity of each cDNA was normalized by GAPDH.

To investigate whether IL-12 plus IL-18-induced IFN-γ transcription requires new protein synthesis, peripheral blood CD4^+^ T cells were incubated either with or without IL-12 plus IL-18 for 4 hours after pretreatment or no pretreatment with cycloheximide (CHX). Total RNAs were isolated and analyzed for IFN-γ message by real-time PCR. As shown in Fig. 4, IL-12 plus IL-18-induced IFN-γ transcript level was enhanced by the pretreatment with CHX (6.8 fold), whereas CHX alone did not have any effect. It has been reported that GADD45β plays an important role in IL-12 plus IL-18-induced IFN-γ expression in *in vitro* differentiated murine Th1 cells by activating p38 MAPK through MEKK4 [3]. In our system, we did not observe any induction of GADD45β and GADD45γ messages upon IL-12 plus IL-18 treatment, although, as reported earlier [3], CHX pretreatment strongly induced IL-12 plus IL-18-activated GADD45β and GADD45γ messages. CHX-induced super-induction has been reported in the case of NFκB-dependent COX-2 transcription, which acts by inhibiting the synthesis of IκB [15]. To determine the role of the NFκB/Rel family of transcription factors in IL-12 plus IL-18-induced IFN-γ transcription, splenic CD4^+^ T cells from wild type and cRel knockout mice were activated with IL-12 plus IL-18 for 24 hours and the IFN-γ mRNA levels were determined by real-time PCR. As shown in Fig. 4B, IL-12 plus IL-18-induced IFN-γ mRNA level was drastically reduced in cRel knockout mice, indicating an involvement of cRel in IL-12 plus IL-18-induced IFN-γ transcription. Next, we wanted to investigate whether NFκB/Rel family transcription factors were also involved in IL-12 plus IL-18-induced IFN-γ transcription in human CD4^+^ T cells. We used IKK-2 inhibitor to block NFκB-mediated gene transcription [16]. As shown in Fig. 4C, IL-12 plus IL-18-induced IFN-γ mRNA level was decreased by IKK-2 inhibitor in a dose-dependent manner, indicating that the NFκB/Rel family transcription factors also play an important role in IL-12 plus IL-18-induced IFN-γ transcription in human CD4^+^ T cells. Collectively, these data suggest the following: first, in resting human peripheral blood CD4^+^ T cells, IL-12 plus IL-18-induced IFN-γ expression is not dependent on new protein synthesis; second, GADD45β and GADD45γ do not appear to play any role in IL-12 plus IL-18-mediated IFN-γ mRNA expression; and third, NFκB/Rel family transcription factors play an important role in IL-12 plus IL-18-induced IFN-γ expression.

### Naïve CD4^+^ T cells, as well as the memory population, respond to IL-12 plus IL-18 in producing IFN-γ

**Figure 5:**
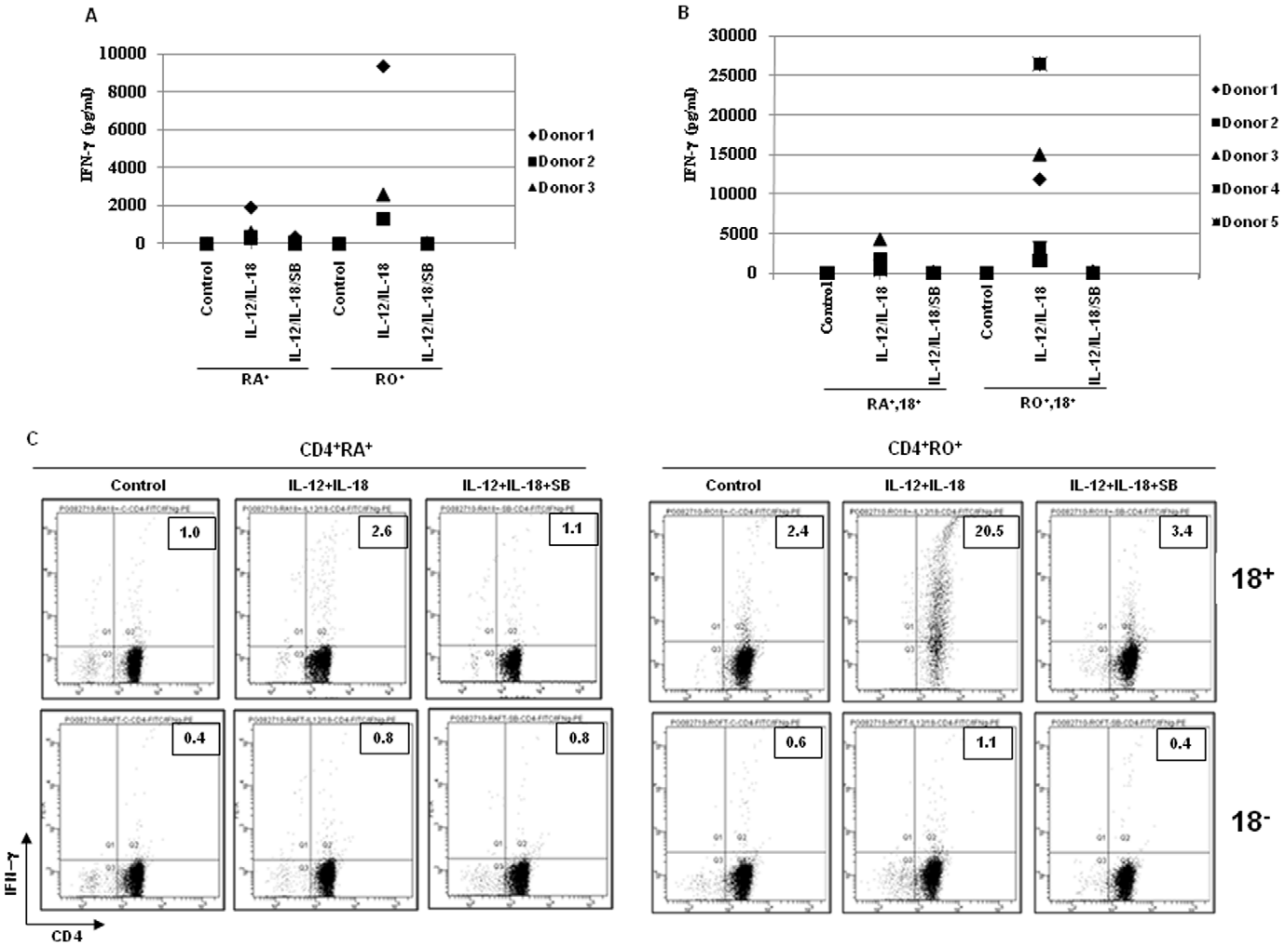
Production of IFN-γ by the sub-population of resting human peripheral blood CD4^+^ T cells after activation with IL-12 plus IL-18. (A) Naïve (RA^+^) and memory (RO^+^) cells isolated from human peripheral blood CD4^+^ T cells were stimulated with IL-12 and IL-18 for 48 hours in the presence or absence of SB203580. Supernatants were analyzed for IFN-γ production. (B) Responsiveness of IL-18Rα^+^ T cells to IL-12 plus IL-18. Freshly isolated IL-18Rα^+^ T cells from naïve (RA^+^18^+^) and memory (RO^+^18^+^) CD4^+^ T cells were treated with IL-12 plus IL-18 for 48 hours in the presence or absence of SB203580. Supernatants were analyzed for IFN-γ production. (C) IL-18Rα^+^ (upper panel, 18^+^) and IL-18Rα^−^ (lower panel, 18^−^) T cells from naïve (CD4^+^RA^+^) and memory (CD4^+^ RO^+^) T cells were treated with IL-12 plus IL-18 for 48 hours in the presence or absence of SB203580, and the cells were then used for intracellular staining for IFN-γ. One representative donor among three is shown here, and data from rest of the donors are shown in Tables 2 & 3.

To investigate whether the responsiveness of CD4^+^ T cells to IL-12 plus IL-18 treatment was unique to either naïve or memory populations, naïve (CD45RA^+^) and memory (CD45RO^+^) cells were purified from resting CD4^+^ T cells by negative selection, and were activated with IL-12 plus IL-18 in the presence or absence of SB203580. Interestingly, both naïve and memory populations were capable of responding to IL-12 plus IL-18 in producing IFN-γ, and as expected, memory populations produced higher amounts of IFN-γ than naïve populations (for three donors ranged from 318 to 1907 pg/ml for naïve, and 1272 to 9368 pg/ml for memory populations, Fig. 5A).

To isolate the CD4^+^ populations responsive to IL-12 plus IL-18, we purified IL-18Rα^+^ cells from both naïve and memory CD4^+^ populations, since this is the receptor chain among others (IL-18Rβ, IL-12Rβ1 and IL-12Rβ2) that has the highest expression levels on resting T cells (data not shown). Isolated IL-18Rα^+^ CD4^+^ T cells were treated with IL-12 plus IL-18 for 48 hours in the presence or absence of SB203580, and the supernatants were assayed for IFN-γ. As shown in Fig. 5B, IL-18Rα^+^ CD4^+^ T cells from both naïve and memory populations produced IFN-γ upon IL-12 plus IL-18 stimulation, and the activation-induced IFN-γ production was blocked by the p38 MAP kinase inhibitor. The amount of IFN-γ produced by five different donors ranged from 443 to 4274 pg/ml for naïve, and 1,591 to 26,496 pg/ml for memory populations.

To determine the percentage of naïve and memory IL-18Rα^+^ CD4^+^ T cells that were capable of producing IFN-γ upon IL-12 plus IL-18 stimulation, purified IL-18Rα^+^ CD4^+^ T cells from naïve (RA^+^18^+^) and memory (RO^+^18^+^) populations along with the corresponding IL-18Rα^−^ cells (RA^+^18^−^/RO^+^18^−^) were activated with IL-12 plus IL-18 in the presence or absence of SB203580 for 48 hours, and the percentage of IFN-γ^+^ CD4^+^ T cells was determined by intracellular staining. As shown in Fig. 5C, 2.6% of naïve IL-18Rα^+^ CD4^+^ T cells and 20.5% of memory IL-18Rα^+^ CD4^+^ T were IFN-γ positive upon activation, and these percentages decreased to the basal level in the presence of SB203580. Moreover, activation-induced IFN-γ^+^ cells were mainly obtained from IL-18Rα^+^ CD4^+^ T cells. The percentage of IFN-γ^+^ cells obtained from three independent donors ranged from 1.5 to 2.6% for naïve population (Table 2), and 15 to 20% for memory population (Table 3). These data indicated that a subset of naïve CD4^+^ T cells are unique in responding synergistically to IL-12 plus IL-18 stimulation in producing IFN-γ.

**Table 2:** Percentage of IFN-γ^+^ cells from CD4^+^ RA^+^IL-18Rα^+^ T cells as determined by intracellular staining.

	Control	IL-12+IL-18*	IL-12+IL-18+SB**
Donor 1	1	2.6	1.1
Donor 2	1.1	1.5	1
Donor 3	0.8	2	1.1

*p = 0.047;

**p = 0.04.

**Table 3:** Percentage of IFN-γ ^+^ cells from CD4^+^ RO^+^IL-18Rα^+^ T cells as determined by intracellular staining.

	Control	IL-12+IL-18*	IL-12+IL-18+SB**
Donor 1	2.4	20.5	3.4
Donor 2	2	15	3.1
Donor 3	1.5	16	2.5

*p = 0.005;

**p = 0.006.

## Discussion

In this report we have shown that a subset of human peripheral blood CD4^+^ T cells can be activated with the combination of IL-12 plus IL-18, but not by either IL-12 or IL-18 alone, to produce IFN-γ in the absence of any antigenic stimulation. A small percentage of resting human peripheral blood CD4^+^ T cells (0.5 to 1.5%) was responsive to IL-12 plus IL-18. Interestingly, both naïve (CD45RA^+^) and memory (CD45RO^+^) CD4^+^ populations that were IL-18Rα^+^ responded to IL-12 plus IL-18 stimulation in producing IFN-γ. The capacity of cytokines to elicit IFN-γ production from resting naïve T cells in an antigen independent fashion indicates a possible role for T cells in innate immunity.

It has been reported previously that IL-12 plus IL-18-induced IFN-γ transcription by *in vitro* differentiated murine Th1 cells requires new protein synthesis and is dependent on GADD45β [3]. In resting human peripheral blood CD4^+^ T cells on the contrary, we show that cycloheximide pretreatment increased IL-12 plus IL-18-induced IFN-γ transcription indicating that new protein synthesis was not required for IL-12 plus IL-18-induced IFN-γ transcription by resting human CD4^+^ T cells. In agreement with this conclusion, IL-12 plus IL-18-induced IFN-γ transcription by resting human CD4^+^ T cells was independent of GADD45β and GADD45γ. This was not due to technical limitations, since, as reported earlier [3], cycloheximide pretreatment strongly induced IL-12 plus IL-18-induced GADD45β and GADD45γ messages. These results are in apparent contradiction with the results reported by Yang et al. [3]. The reason for the discrepancies may be due to the fact that Yang et al. have used *in vitro* differentiated murine Th1 cells, whereas freshly isolated human peripheral blood T cells were used in our study. It is important to note that in spite of the apparent lack of GADD45β involvement in human CD4^+^ T cells, like the murine system [3], IL-12 plus IL-18-induced IFN-γ production by human CD4^+^ T cells was dependent on p38 MAP kinase. This indicates that the activation of p38 MAP kinase by IL-12 plus IL-18 in human CD4^+^ T cells involves a GADD45β-independent pathway. We have also shown here that cRel, an NFκB/Rel family transcription factor, plays an important role in IL-12 plus IL-18-induced IFN-γ transcription. Regarding the signaling mechanism, we have observed a synergy between IL-12 and IL-18 stimulation with regards to Stat4 phosphorylation. The signaling synergy was observed as early as 1 hour, which reached the peak by 6 hours, and persisted for at least 24 hours. More in-depth signaling mechanisms involved in the IL-12 plus IL-18-mediated IFN-γ production by naïve human peripheral blood T cells is currently under investigation.

The *in vivo* physiological role of IL-12 plus IL-18-mediated IFN-γ production by naïve CD4^+^ T cells in the absence of antigen is not known. This pathway may play an important role in innate immunity as a rapid responder to pathogens in the absence of cognate antigen recognition. In accordance with this hypothesis, Berg et al., have shown a contribution of memory murine CD8^+^ T cells to innate immunity in protection against *Listeria monocytogenes* in the absence of cognate antigen by rapidly producing IFN-γ in response to IL-12 and IL-18 [17]. Human CD8^+^ memory T cell subsets have also been shown to respond vigorously to IL-12 plus IL-18 in the presence of IL-15 by producing IFN-γ [18]. Recently, it has been shown that a subset of resting human effector-memory T helper cells can be induced to produce IFN-γ by IL-12 plus IL-18 in conjunction with cytokines signaling via the IL-2R common γ-chain, and the authors have provided rationale for the involvement of effector-memory T helper cells in chronic autoimmune inflammation [19]. The antigen-independent cytokine-mediated pathway of IFN-γ production by T cells may also be involved in other pathological conditions [20]. It has been shown that TL1A (TNF-like cytokine) synergizes with IL-12 plus IL-18 in inducing IFN-γ by CD4^+^ T cells [21], [22], and TL1A production by immune complex-stimulated monocytes may be involved in the pathogenesis of rheumatoid arthritis [23]. This cell subset may also be involved in amplifying an antigen-driven T cell immune response in that IL-12 and IL-18 are both produced in the course of such T-cell receptor-driven processes. Since we have shown here that the cytokine-induced pathway for IFN-γ production is blocked by rapamycin, mTOR may be a therapeutic target in the treatment of autoimmune diseases characterized by IFN-γ overexpression. The existence of a pathway of naïve T cell activation in the absence of antigen may play a role in a wide range of immunological conditions.

## Supporting Information

Figure S1 **Human peripheral blood CD4^+^ T cells from two independent donors were activated with either anti-CD3 plus anti-CD28 or IL-12 plus IL-18 in the presence or absence of cyclosporin A for 24 hours and the supernatants were analyzed for IFN-γ production.** Results from two independent donors are shown here.
(TIF)

Figure S2 **Freshly isolated human peripheral blood CD4^+^ T cells were stimulated with the indicated concentrations of IL-12/or IL-18 for 24 hours and the supernatants were analyzed for IFN-γ production.**
(TIF)

Figure S3 **Freshly isolated human peripheral blood CD4^+^ T cells from two independent donors were stimulated with IL-12 (1 ng/ml)/IL-18 (40 ng/ml) for various periods of times to measure IFN-γ production.**
(TIF)

Figure S4 **Freshly isolated human peripheral blood CD4^+^ T cells from two independent donors were cultured either in the medium alone or in the presence of IL-12 plus IL-18 for various periods of times.** At each time point, cell viability was determined by trypan blue staining, and cell numbers were determined by cell count.
(TIF)
