# Supplementary figures and images for: Antigen-Independent IFN-γ Production by Human Naïve CD4+ T Cells Activated by IL-12 Plus IL-18
Source: PLoS One. 2011 May 10;6(5):e18553. doi: 10.1371/journal.pone.0018553 (PMC3091853; doi:10.1371/journal.pone.0018553)

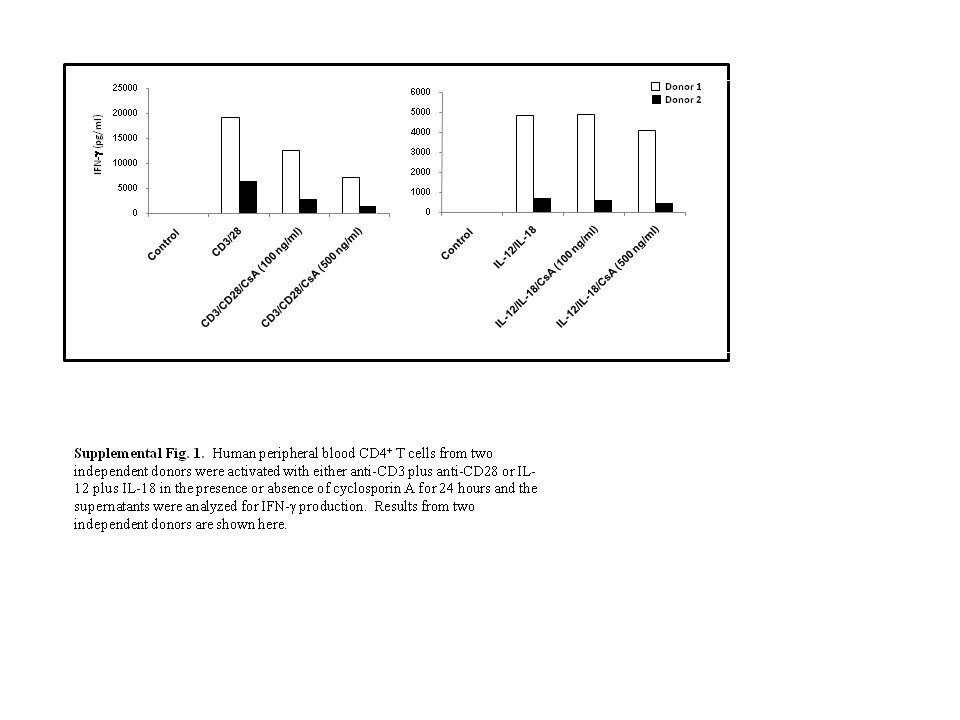

Supplement: Figure S1 — Human peripheral blood CD4+ T cells from two independent donors were activated with either anti-CD3 plus anti-CD28 or IL-12 plus IL-18 in the presence or absence of cyclosporin A for 24 hours and the supernatants were analyzed for IFN-γ production. Results from two independent donors are shown here. (TIF) [file pone.0018553.s001.tif]

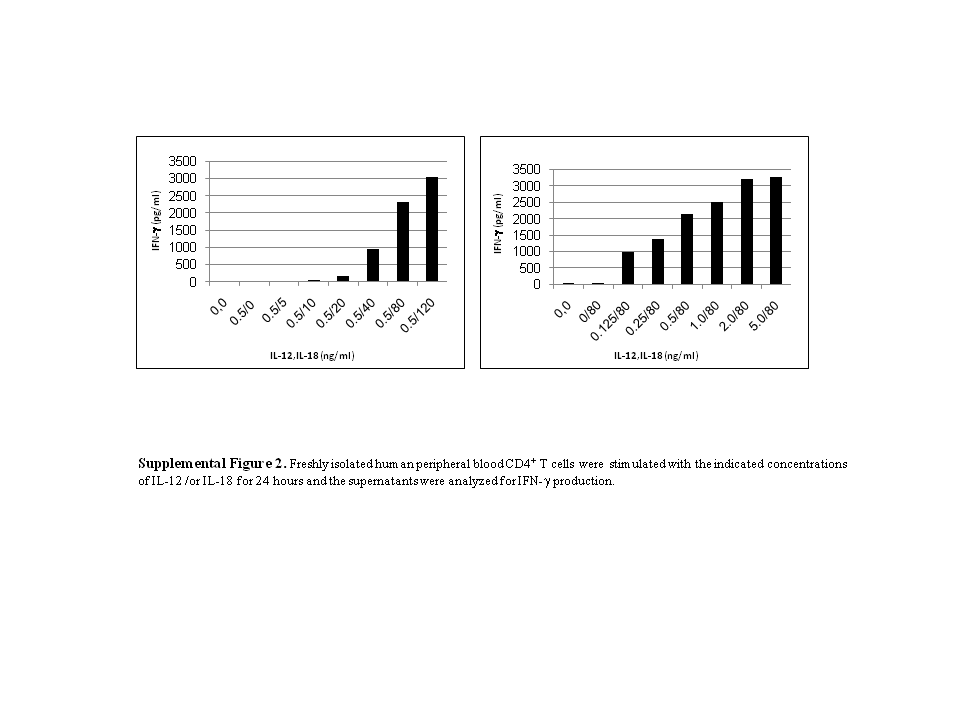

Supplement: Figure S2 — Freshly isolated human peripheral blood CD4+ T cells were stimulated with the indicated concentrations of IL-12/or IL-18 for 24 hours and the supernatants were analyzed for IFN-γ production. (TIF) [file pone.0018553.s002.tif]

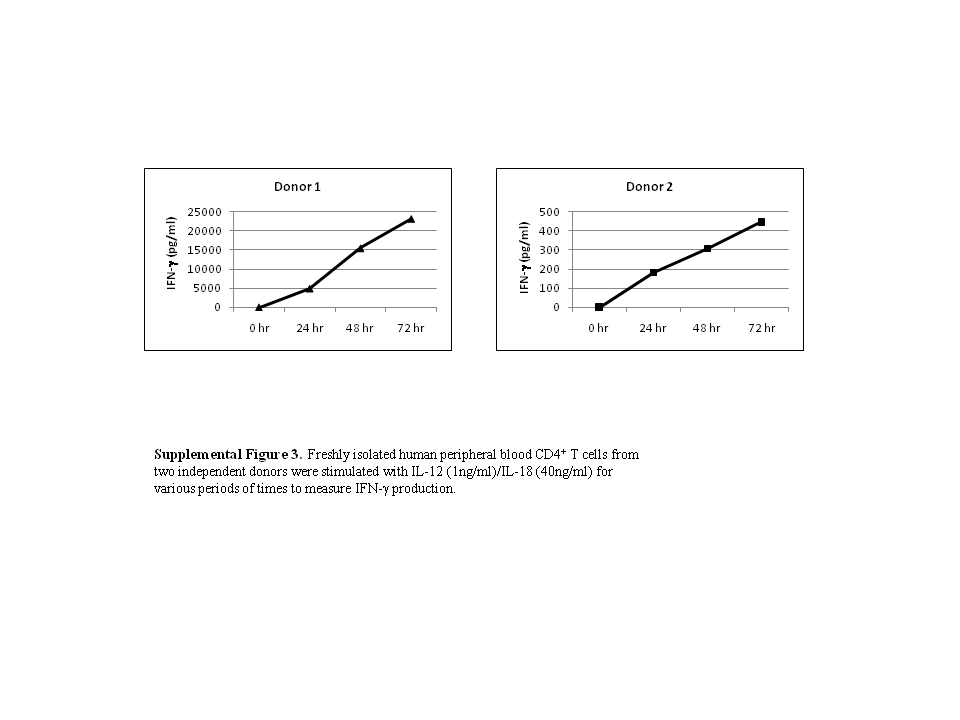

Supplement: Figure S3 — Freshly isolated human peripheral blood CD4+ T cells from two independent donors were stimulated with IL-12 (1 ng/ml)/IL-18 (40 ng/ml) for various periods of times to measure IFN-γ production. (TIF) [file pone.0018553.s003.tif]

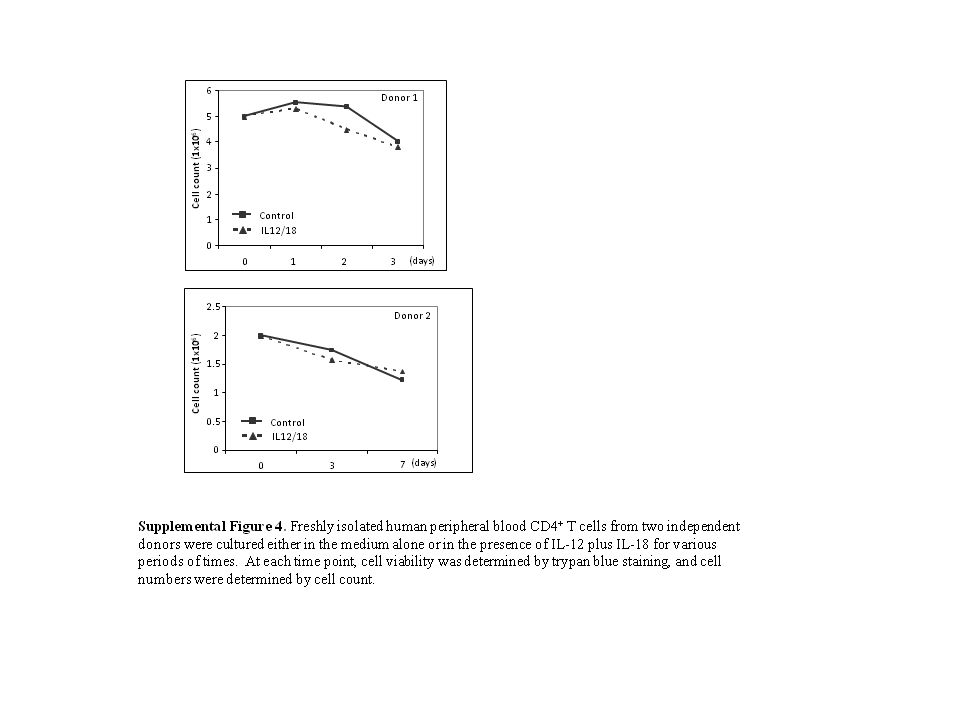

Supplement: Figure S4 — Freshly isolated human peripheral blood CD4+ T cells from two independent donors were cultured either in the medium alone or in the presence of IL-12 plus IL-18 for various periods of times. At each time point, cell viability was determined by trypan blue staining, and cell numbers were determined by cell count. (TIF) [file pone.0018553.s004.tif]
